# The influence of language of instruction in the facilitation of academic activities: Nurse educators’ experiences

**DOI:** 10.4102/hsag.v24i0.1261

**Published:** 2019-08-27

**Authors:** Gugu Ndawo

**Affiliations:** 1 Department of Nursing, Faculty of Health Sciences, University of Johannesburg, Johannesburg, South Africa

**Keywords:** language of instruction (LOI), nurse educator, proficiency, academic activities, experiences, LOI incompetence

## Abstract

**Background:**

Learners in most South African higher education institutions are taught mainly through English for most of their academic lives, yet many of them enter these institutions with poor proficiency in this language of instruction (LOI).

**Aims:**

The purpose of this article was to describe the experiences of nurse educators teaching in a 4-year comprehensive nursing diploma programme regarding the use of English as the LOI during academic activities.

**Setting:**

The nursing college under study offers a 4-year comprehensive nursing diploma programme as well as post-basic diploma qualifications such as Primary Health and Midwifery Nursing Science and is situated in Gauteng, South Africa.

**Methods:**

Twenty nurse educators were purposively sampled for in-depth individual interviews until data saturation and were requested to participate in the study. Tesch’s protocol of qualitative data analysis was used and the themes that emerged were confirmed by an independent coder. Trustworthiness was ensured, and ethical considerations were adhered to.

**Results:**

It emerged that English language incompetence (1) undermines learners’ self-esteem; (2) hinders critical, reflective and creative thinking; (3) renders understanding difficult and that (4) nurse educators’ incompetence in LOI hinders meaningful teaching.

**Conclusions:**

Recommendations were made to improve the use of the LOI because through language interdisciplinary knowledge and understanding are integrated, thus ultimately providing patients with comprehensive, holistic and transcultural healthcare.

## Introduction

South Africa is one of the most culturally diverse and multilingual countries with 11 official languages that enjoy the protection of the Constitution (Department of Justice and Constitutional Development 1996, December). Three-quarters of the South African population speak isiZulu and isiXhosa, whereas English, although used in the facilitation of academic activities in institutions of learning, is home language to only 9.6% of South Africans. Its fluency and proficiency among this population and other indigenous South African populations, namely Sepedi, Sesotho, Setswana, siSwati, Tshivenda, Xitsonga and isiNdebele, is limited (Coffi 2017). In spite of this, most South African higher education institutions teach mainly in English. This was also true for the nurse educators and learners at the nursing college in this study where English was the language of instruction (LOI) even though it was not the mother tongue for the majority of them. They are thus English-as-a-Second Language (ESL) individuals (Kotzé, Van Der Westhuizen & Barnard 2017).

Globally, English is considered an international language (Abdallah & Gabr 2014), the language of commerce, technology, education and training which has become indispensable for economic emancipation (Coffi 2017). A language of written laws (Joyce-Gibbons et al. 2018) that is ‘rich in literature, humanistic and scientific approach and technical information’ (Roy 2018:240) and viewed as the only language in which knowledge is useful (Ramachandran & Rauh 2016). However, according to Qorro (2006:4), these may be among many ‘good reasons for teaching English, but not for teaching in English’ because as a LOI to ESL learners it has huge impact on the quality of their education. In spite of this argument, nothing has changed; learners are still taught in English in many higher education institutions globally (Kotzé et al. 2017; Qorro 2013).

When nurse educators are proficient in the LOI, they use proper communication that facilitates deep learning, firm understanding of scientific nursing concepts and content knowledge, thus facilitating cognitive growth of learners. In facilitating understanding of the subject matter, these educators confidently use appropriate innovative and creative pedagogical and assessment strategies that assist in the development of learners’ own innovative and creative skills needed in the 21st century (Joyce-Gibbons et al. 2018; Purwaningsih 2018). That way they assist learners in developing a skill of applying the newly constructed and reconstructed knowledge in different healthcare contexts (Qorro 2013). These live, fluent educators stimulate interest and curiosity in their learners and encourage their engagement in critical discourse and conversations on more complex learning content to develop their higher order thinking skills. They encourage and embrace learners’ challenging critical questions that they use to challenge their learners back (Richards 2017). Moreover, such educators create a learning environment that is conducive to meaningful learning because they possess knowledge and understanding of the importance of uniformed culture and language in academic achievement.

They encourage learners to make mistakes and take academic risks while providing scaffolding, mentoring, coaching and guidance (Kotzé et al. 2017). These nurse educators engage in the scholarships of discovery, integration, application and teaching and learning, which have high demand on their own higher order thinking skills such as critical, creative, reflective, conceptualisation and integration. They understand that such skills are highly facilitated by, and require, the use of language of communication for knowledge generation in improving the practice of teaching, using evidence-based research and applying it to advance learners’ academic development and success as well as their own (Bruce & Klopper 2017). Through confidence they engage in continuous professional development that ensures learners’ academic success through the use of evidence-based innovative academic activities (Richards 2017). Therefore, in nursing education learners successfully learn the science and art of nursing.

Learners, in contrast, use language and culture to identify with others, while their socio-political factors influence their learning and learning perceptions. This means that when learners meet others who share significant similar characteristics with themselves such as language and culture, they willingly and meaningfully engage in academic activities. Likewise, when such characteristics are absent, they are reluctant and unenthusiastic to engage and make meaningful social links for diverse constructive learning, thus resulting in lack of learner’s adaptability (Dogan 2013). Meaningful interaction with others using language that is understood by all results in cognitive development through critical discourse that requires critical thinking about the matter at hand (Vygotsky in Kotzé et al. 2017). This means that proficiency in the LOI results in learner academic improvement by encouraging dynamic meaningful engagement with significant, more-abled others, which learners use to their own advantage as they identify and seize learning opportunities that assist them in creating their own new meaning (Miti 2017). Thus, they become creative to ensure that they learn meaningfully and succeed academically.

However, nurse educators and learners at this nursing college were multilingual and as a group did not share similar characteristics such as language and culture. They were compelled to uphold each other’s constitutional rights within the dynamic learning environment in which they interacted because they understood that lack of such interaction had serious and dire consequences for their learning (Chabeli, Malesela & Rasepae 2014). As a result, English as a LOI prevailed in spite of lack of proficiency. According to the sixth critical cross-field outcome of the South African Qualifications Authority (SAQA) (1995, October), a learner should be culturally and aesthetically sensitive across a range of social contexts such as language. To develop such sensitivity, they must demonstrate an understanding, willingness and commitment to engage with other learners in the language that is understood by all. Because English was a LOI that steered every major academic and social interaction in this nursing college, it was important to explore the nurse educators’ experiences of how it influenced the facilitation of their teaching, learning and assessment activities.

## Problem statement

English is the LOI at the nursing college in this study, yet some nurse educators and the majority of their learners are ESL individuals and not proficient in it. The non-proficiency constitutes a language barrier that generates negative cognitive and emotional responses for both ESL educators and learners (Kotzé et al. 2017). For example, ESL educators with limited proficiency of the LOI have difficulty in giving clear, proper instructions to learners that guide meaningful learning. They shy away from and do not allow learners to ask questions as well as fail to reply and respond to their questions or remarks effectively. They lack authority and self-confidence in the classroom and as a result their performance, which includes teaching, assessment, modelling and influencing, is negatively affected (Richards 2017).

In addition, ESL learners who lack proficiency in the LOI learn that they are unintelligent, less capable than their fluent counterparts and therefore unlikely to succeed. They have a limited grasp of the subject matter because they do not engage with the learning content at a much deeper level but rote learn to pass the examination (Evans & Morrison 2017). This practice, in turn, does little to improve their knowledge construction and acquisition, which result in lack of meaning making (Joyce-Gibbons et al. 2018). English-as-a-Second Language learners have challenges to adapt to the higher education demands that can be a challenge in developing their academic literacy, problem-solving, rational decision-making and communications skills and encouraging constructive engagement in their learning processes. They perform poorly in their academic studies as they experience difficulty with coping using a language in which they are not conversant (Vuzo 2018). As a result, they may take longer to complete and more likely to drop out of the nursing programme.

The researcher could not find any evidence of existing research that explored the issue of LOI at this nursing college and therefore asked the following question: What are nurse educators’ experiences on the influence of LOI in the facilitation of academic activities?

## Research purpose

The purpose of this study was to gain an in-depth understanding of the experiences of nurse educators on how English as a LOI influences the facilitation of academic activities.

### Research design and method

#### Research design

A qualitative, phenomenological and contextual research design was used (Gray, Grove & Sutherland 2017) to systematically and subjectively explore and describe the nurse educators’ experiences regarding the influence of English as a LOI in the facilitation of academic activities. Qualitative is a research approach used to describe the lived experiences of the participants from their own perspective (Gray et al. 2017). A descriptive phenomenology was used in the exploration, analysis and in-depth description of the lived experiences of nurse educators on the influence of LOI in the facilitation of academic activities. This means that nurse educators’ experiences were conveyed and described precisely as understood by them. Objectivity was maintained by the researcher through putting aside their own preconceptions and opinions about the phenomenon at hand, thus limiting biasness (Creswell & Poth 2018). In addition, the researcher held meaningful discussions with academic peers, study supervisor and independent coder on the collected data to ensure honest data analysis and interpretation of findings. These individuals also assisted the researcher with self-detachment and bracketing by providing thought-provoking reflections on the reflexive diary that the researcher kept (Ndawo 2017).

#### Population and sampling

The target population included nurse educators who were teaching learners registered in the 4-year diploma programme leading to registration as a nurse (general, psychiatric and community) and midwife, as stipulated by the South African Nursing Council (SANC) Regulation 425 of 1985 (as amended). They possessed an additional qualification in nursing education according to SANC Regulation 118 of 1987 (as amended). A purposive sample was determined by data saturation, which was reached at the 20th participant when no new information emerged (Gray et al. 2017). The participants were viewed as having rich data because of the 20 nurse educators, 18 were Africans and two were of mixed race and sampled under the following criteria.

Inclusion criteria:

- They willingly consented to participate in the study.
- English was a second language for all nurse educators.
- They used English as a LOI in their daily academic activities, which included teaching, learning and assessment.
- They were never schooled in a multi-racial background, thus were having rich data to best answer the research question.
- They have been teaching at this nursing college using English as a LOI between the periods of 2–19 years.

Exclusion criteria:

- English-as-a-First Language (EFL) nurse educators were excluded in the study.
- English-as-a-Second Language nurse educators who were not involved in academic activities of the learners registered in the 4-year diploma programme leading to registration as a nurse (general, psychiatric and community) and midwife (SANC 1985).

#### Data collection

The researcher collected the data by means of in-depth individual interviews because this method is organised around specific areas of interest while allowing considerable flexibility in scope, depth, accessibility and time for participants in telling their stories (Gray et al. 2017). The question asked was: In your experience, how does English as a language of instruction influence the facilitation of teaching, learning and assessment? Facilitative communication clarification techniques such as probing, paraphrasing and summarising were used for detailed exploration of data (Murphy & Dillon 2015). Field notes that described the communication dynamics were taken and all interviews were audio-tape recorded with the permission of the participants to increase the credibility of the collected data through providing accurate material for intensive analysis (Gray et al. 2017).

#### Data analysis

Data were transcribed verbatim immediately following each interview and analysed manually together with the field notes using Tesch’s protocol of qualitative data analysis (Creswell & Poth 2018). The researcher read and reread all interviews, looking for underlying meaning in the data. Similar topics were grouped together and themes were constructed. The data were recoded to ensure accuracy. A consensus meeting with an independent coder was held and an agreement on the independently identified themes that emerged was reached. Follow-up interviews with 11 participants were also conducted to verify the accuracy of the verbatim transcribed interviews and the truth value of the identified themes (Gray et al. 2017). The researcher stopped at the 11th follow-up interview because all the 11 participants who were consecutively approached indicated that the transcribed notes were the true accounts and reflection of their lived experiences as verbalised by them.

#### Ethical considerations

Permission to conduct the research was requested and obtained from the University of Johannesburg, Faculty of Health Science’s Research Ethics Committee (AEC 43-01-2012), the Department of Health and the nursing college. Written informed consents to participate and permission to record the interviews were obtained after the purpose of the study was thoroughly explained to the participants and their questions truthfully answered. The participants were informed that participation was voluntary and that they could withdraw from the study at any time without penalties (Dhai & McQuoid-Mason 2011). Pseudonyms were used during interviews as well as in the subsequent reporting, thereby ensuring the anonymity of the data.

#### Trustworthiness

According to Lincoln and Guba (1985), trustworthiness is a method used to ensure rigor in qualitative research without losing relevance. Table 1 demonstrates how the four criteria of trustworthiness, credibility, transferability, dependability and confirmability were applied.

**TABLE 1:** Trustworthiness strategies applied in the study.

Strategy	Criterion	Application
Credibility	Prolonged engagement	The researcher spent as much time as possible with participants to build trust and rapport, and immersed in the data by listening to the tapes repeatedly to familiarise self with the collected data before developing themes.
	Reflexivity	The researcher constantly reflected on the collected data and strove to avoid possible biases so not to influence the research findings. A reflexive diary was kept by the researcher at all times and its content was later discussed with academic peers, study supervisor and independent coder.
	Member checking	Follow-up interviews with 11 participants were conducted to verify the accuracy of the verbatim transcribed interviews and the truth value of the identified themes. Findings were reported as nurse educators’ true reflection of their *experiences*.
Transferability	Sample selection	The sample of participants was purposively selected. The research design and method were adequately described.
Dependability	Dense description	Dependability was ensured through a dense description of the data and the use of an independent coder to independently analyse the data using Tesch’s protocol.
	Stepwise replication	The research process was described in such a way that other researchers can follow similar steps and in each interview the same question was posed to all participants.
Confirmability	Confirmability audit	The results and findings including records such as field notes, audio-tape recordings, transcription notes, coding details and a proposal were compiled and kept to demonstrate what occurred during the research process.

## Findings

The English language as a LOI was experienced by nurse educators as having a negative influence in the facilitation of academic activities that created a barrier that impeded meaningful learning. The negative influence experienced was evident in the four main themes that emerged which were (1) nurse educators’ incompetence in LOI hindered meaningful teaching. Language incompetence (2) rendered understanding difficult; (3) hindered critical, reflective and creative thinking; and (4) undermined learners’ self-esteem. Below is the display of verbatim accounts of each theme and later the discussion of findings.

### Theme 1: Nurse educators’ incompetence in language of instruction hindered meaningful teaching

All participants stated that when they have limited command of the LOI, their teaching performance and subsequent learning were impeded. Of great importance, this teaching and learning impediment came at a hefty price of resorting to inadequate teaching and assessment strategies. This is evident by the following quotes:

‘When I teach in English I do not come across as a person who understands what she is teaching to students, it is quite embarrassing. So I stick to what I can say regarding learning content and only ask few questions that will not warrant me to explain much ….’ (Participant 12, African, 55 years)

‘… I usually resort to asking basic questions that do not require me to elaborate on the content … **(**pause) … not being fluent … they (students) don’t learn much … (Sounding concerned).’ (Participant 17, African, 48 years)

‘… and they (students) refer to me now as ‘Ma’am Compact’ of which I meant “Compat” for “Compatibility” … It is quite embarrassing because now I’m called by a mistake and also I know that the lecture shifted to my language mistakes … and no longer to learning.’ (Participant 10, African, 39 years)

But one participant also clarified that:

‘… to some extent, because I have been teaching for a while, I have some degree of proficiency but I’m certainly not English, I still experience those challenging moments that limit my teaching ….’ (Participant 4, Mixed Race, 43 years)

The participants also stated that they usually use lecture method and easy assessment methods that do not require time and energy for application and explanations. They also acknowledged the fact that this was limiting in their teaching. The participants said in this regard:

‘I rely on the fact that I’m not here to teach English but a nursing subject … but that in itself is very limiting in one’s teaching and own learning ….’ (Participant 17, African, 48 years)

‘I can hardly, effectively and meaningfully critique student assignments because it is difficult to apply my mind to those minority who used their creative thinking so I resort to multiple-choice questions and those of “true or false” and always use textbook memoranda to cover my back and ensure fairness ….’ (Participant 9, African, 65 years)

### Theme 2: Language incompetence rendered understanding difficult

Most participants stated that their frustrations were related to learners’ understanding and that it was difficult for learners to think of their own examples to better their understanding. Some participants said:

‘… I find myself repeatedly saying “But we did this yesterday!!!” Yet yesterday’s content wasn’t understood and they (students) were afraid to admit that.’ (Sounding frustrated) (Participant 6, African, 61 years)

‘… they (students) stare at me without understanding what I’m saying to them. So, usually they recite even my own examples that I have made to them to try and clarify the content. They fail to come up with their different creative examples ….’ (Participant 19, African, 63 years)

Most participants also pointed out that LOI proficiency in nursing education is important as learners needed to understand the nursing terminology associated with each specific nursing subject, yet this was not the case within their teaching and learning environment. Some participants stated that:

‘Students need more than just understanding English, they must also understand the … nursing terminology that is required to become a professional nurse.’ (Participant 13, African, 57 years)

‘To create understanding the student must make mental pictures for themselves so they must think in a language and subject … and appropriately use the nursing jargon so that they see the connection …’ (Participant 1, African, 39 years)

### Theme 3: Language incompetence hindered critical, reflective and creative thinking

The findings of the study revealed that a lack of command of the LOI was experienced by the participants as a hindrance to critical, reflective and creative thinking of learners. Some participants echoed that:

‘I have noted that it is a big challenge for the students to argue in English and put their viewpoints across in such a way that they are understood.’ (Participant 5, African, 45 years)

‘… students debate and argue very well in their own languages and can reason things out but they can’t achieve that when they can hardly think and respond in a language that is not theirs ….’ (Participant 18, African, 62 years)

The participants also experienced that lack of the command of the LOI hindered the process of critical thinking development in learners. They said:

‘… this (lack of command of LOI) also hinders students’ curiosity, inquisitiveness and as a result there is no critical thinking that is being developed during learning.’ (Participant 4, Mixed Race, 43 years)

‘… students can’t reason properly and in a systematic manner ….’ (Participant 18, African, 62 years)

‘It is quite surprising to read what they (students) have written in their reflective journals and actually listening to what they narrate about the same experience because it’s not the same … (pause) … They can write very creatively in their own language yet cannot narrate equally in English.’ (Participant 5, African, 45 years)

### Theme 4: Language incompetence undermined learners’ self-esteem

Most participants felt that ESL learner’s self-esteem was negatively affected if they could not accurately and proficiently express their knowledge, experiences, thoughts, feelings and emotions about the learning content. The participants expressed that:

‘Students struggle to speak English during interactive sessions. They become anxious and fidgety … it becomes embarrassing for them especially during class discussions.’ (Participant 8, African, 48 years)

‘Argumentation is an effective strategy to facilitate logical thinking but for students to engage in it they need loads of self-esteem which they don’t have when they don’t know English well.’ (Participant 4, Mixed Race, 43 years)

The low self-esteem was evident in the lack of learners’ participation in classroom activities. Other participants added that:

‘They (students) will rather keep quiet than interact in broken English in front of others.’ (Participant 11, African, 39 years)

‘Majority of students are silent and passive in class in spite of the information they have because of lack of command of the language. They don’t want to be seen as unintelligent because they can’t communicate in English.’ (Participant 16, African, 40 years)

‘… they nervously say “Ma’am, may I please rather say this in my language?”.’ (Participant 17, African, 48 years)

## Discussion of findings

### Theme 1: Nurse educators’ incompetence in language of instruction hindered meaningful teaching

The study findings highlighted that the participants were, to some extent, also challenged with the use of English as a LOI in their learning environments – a finding experienced by them as a hindrance to meaningful teaching and subsequently to learning. This resulted in them relying on traditional instructional methods that did not stimulate learners’ critical thinking nor refine their logical thinking, but encouraged regurgitation of facts that left learners disinclined to seek clarity. Similarly, Richards (2017) found that many ESL educators did not use innovative learner-centred pedagogical strategies such as inquiry-based argumentation and technology within their learning environments for fear of being unable to clearly articulate their own thinking and relate to their real-life experiences owing to lack of proficiency in LOI. They experienced difficulty in explaining the learning content in a way that learners can understand because they also struggled to make their own meaning using LOI, which stifled their teaching and as a consequence stifled learners’ meaningful learning.

Some participants went to an extent of asking few basic, lower order thinking questions that did not require them to elaborate on the learning content. According to Kivunja (2015), challenging and thought-provoking questions such as Socratic questioning and De Bono’s six thinking hats equip learners with skills needed for clinical reasoning and judgement, complex problem-solving and rational decision-making, higher order thinking skills that are indispensable in providing exceptional nursing care. Therefore, when ESL nurse educators do not challenge learners using these pedagogical strategies because they themselves are challenged by the LOI, meaningful teaching and learning is hindered. Learners are left on their own to develop and acquire higher order thinking skills, an exercise that is unattainable because learners find these skills most difficult to acquire on their own (Starr-Glass 2013).

Some participants also stated that they had difficulty to teach and facilitate learning in a manner that was free of errors. According to Purwaningsih (2018) and Sharif (2013), educator’s lack of proficiency in the LOI may lead to a contortion of the factual truth of the content that learners learn. Their flawed pronunciation might lead to improper meaning that might have a negative impact on learners’ future professional careers and lifelong learning (Purwaningsih 2018). They may experience difficulty in clarifying abstract concepts by relating such concepts to real-life examples and narratives to provide greater insights for learners on the subject. The participants also experienced doubt and loss of self-confidence. Such loss stops ESL nurse educators from demonstrating and modelling critical thinking processes to learners such as thinking aloud while solving a real-life patient’s problem. Joyce-Gibbons et al. (2018) and Kirui, Osman and Naisujaki (2017) state that to cope and protect their image, ESL educators opt to use safe talk that is highly limited to teaching only the learning content at hand which, sadly, limits learners’ engagement in meaningful learning and construction of own knowledge within a learning environment.

There was an intentional neglect of learners’ written language, as stated by the participants. Consequently, there is a missed opportunity to facilitate learning of the LOI because nurse educators only concentrated on the learning content as they were not assigned to teach English but a nursing subject. They also admitted that this practice is limiting in one’s own teaching and learning. However, Sharif (2013) contend that such neglect may indirectly inform learners that their English is correct and as a result they may not realise that their language skills need to be improved. Furthermore, both ESL educators and learners may be left ill prepared to critically analyse, reason, interpret, conceptualise, evaluate and apply judgement to the learning content. Leaving both ESL educators and learners disadvantaged because they are challenged in utilising such skills to become lifelong learners, ensure their academic success and improve their careers as scholars and professional nurses.

Some participants conceded that they neglected to critique learners’ work because of difficulty in applying their minds to some learners’ creative work. Henderson et al. (2015:1669) state that critiquing is crucial because it makes learners’ thinking evident through identifying unsound reasoning and essentially providing ‘constructive feedback about the nature of learner’s understanding’ to the critic. Thus, critiquing improves learners’ understanding of the learning content. Moreover, it stimulates learners’ critical reflection of what they have learnt, thus promoting meaningful engagement with the learning content. When nurse educators do not critique learners’ work, they fail to clarify learners’ misconceptions and misunderstandings that might lead to academic failure. Critiquing is a challenging, time-consuming exercise that, if not performed, leaves educators with sufficient time to progress through the syllabus smoothly while superseding deep learning (Joyce-Gibbons et al. 2018). It is worth noting that all participants knew that their lack of proficiency in LOI stifled effective teaching and assessment, which negatively affected meaningful learning.

### Theme 2: Language incompetence rendered understanding difficult

The participants shared their frustration that the use of LOI in which their ESL learners lacked proficiency made understanding of the learning content very difficult. This was evident when some ESL learners failed to come up with their own thought of examples based on their life experiences and understandings, but recited the examples that were previously made by the participants in class. According to Chabeli and Muller (2004:42), understanding occurs because the ‘representational properties of the words facilitate the transformational aspects of thought’, while verbalising refines the products of thought involved – skills which ESL learners do not possess. According to Richards (2017), ESL learners regurgitate facts because without command of the spoken language, understanding and thinking about such facts is not possible because these cognitive skills are enhanced by linguistic clarity within a specific context. Therefore, these regurgitated facts remain isolated and cannot be used in the recreation and creation of new knowledge through integration with prior knowledge and as a result cannot be internalised for future use in new challenging learning and clinical situations. Furthermore, such facts develop into inert and impractical knowledge that has no meaning and relevance to learners and their patients (Ndawo 2017) – a shortfall that results in a critical learning impediment (Scherer 2016). For this reason, lack of understanding because of LOI incompetence and not lack of knowledge is associated with academic failure in spite of ESL learners’ high levels of engagement with the learning content (Kirui et al. 2017).

The participants also noted that it was difficult for ESL learners to relate to the science of a nursing subject and as a result learners often experienced stress and anxiety. According to Sharif (2013), such emotions could result in ESL learners even failing the subjects as a consequence. Failure is associated with a lack of command of the LOI resulting from learners’ inability to communicate conceptual knowledge of a subject. Furthermore, their cognitive academic language proficiency (CALP) is not yet developed which is important for analysing, evaluating, inferring and interpreting terminology and more complex, advanced and abstract information associated with such subject. Scherer (2016) concurs that when ESL learners face challenges with mere basic interpersonal communication skills it would be impossible that they would not experience difficulty in relating their understanding to the science of the nursing subject. The use of foreign terminology with each different nursing subject affects their learning capability, which, in turn, increases their risk of academic failure.

According to some participants, ESL learners needed to construct mental pictures for themselves to create understanding. However, language affects mental activities of its users (Vygotsky in Kotzé et al. 2017) and construction of knowledge and understanding occurs by building on a cognitive structure and through creating connections out of an experience (Ausubel in Jain 2014). But when ESL learners lack proficiency in the LOI, they fail to make mental pictures and create connections based on experience. They are left on their own to try and figure out the meaning of the learning content, the nursing and medical terminology in particular. They fail to elaborate on and interpret the new information, but simply process it because they lack command of the LOI. They also fail to construct their own understanding; therefore, deep meaningful learning and academic growth are stifled (Scherer 2016). This results in failure to adapt, integrate and apply the taught information to new, complex and challenging clinical situations, yet adaptation, integration and application of knowledge are the basis for developing critical skills in nursing (Ndawo 2017). Understanding may be the lower order thinking skill, but it is very important in forming the foundation for higher order thinking such as analysis, evaluation, problem-solving, rational decision-making, critical, reflective and creative skills.

### Theme 3: Language incompetence hindered critical, reflective and creative thinking

The participants stated that when learners lack command of the LOI, they had difficulty engaging in learning activities that stimulated and developed higher order thinking skills such as argumentation and debate. This was a result of that learners had challenges in expressing themselves in a way that they were understood by others. Language empowers higher order thinking skills and therefore enhances comprehension, meaning and cognitive development through meaningful interactions with more knowledgeable others (Vygotsky in Naude, Van Den Bergh & Kruger 2014). English-as-a-Second Language learners who are not fluent in the LOI are disinclined to engage in dialectic dialogic activities such as argumentation and debate within the community of learners because of their inability to make sense of their worlds, explain, argue and clearly articulate their knowledge, thoughts, emotions, feelings and innovative ideas. Their lack of command of the LOI also quells their intellectual curiosity and inquisitiveness, which are needed for the development of critical thinking (Kotzé et al. 2017), as also noted by the participants. Lack of curiosity has a negative impact on the desire of ESL learners to learn and enhance their knowledge. They lack the ability to use the state of wonder and lack interest in search of their own information for deeper inquiry and clarity while being open to diverse multiple perspectives. Moreover, they fail to discover new insights such as counterarguments, which further stifles the development of higher order thinking skills and meaningful learning.

According to the participants, learners had difficulty in using logical reasoning during interactive group activities, argumentation and debate. Logical reasoning is a pillar of higher order thinking skills that is enriched by linguistic clarity and can be employed in formulating and justifying arguments. But when the command of reasoning language is lacking, ESL learners cannot organise their thinking, convey and share complex, advanced and abstract information through structured critical discourse (Naude et al. 2014). This means that their logical reasoning is negatively affected and their critical thinking muddled. They, thus, find it difficult to make rational clinical judgements and decisions, unable to solve real-life complex problems and innovatively create real-life solutions (Kivunja 2015).

English-as-a-Second Language learners in this study had difficulty narrating their academic and clinical experiences, which were written in their reflective journals in the LOI, as noted by the participants. Constructing critical reflective activities using technology such as electronic Journals, electronic Portfolios, blogs and discussion boards is important in any science and discipline as these activities assist with construction of meaningful learning (Frank 2015). Lack of command of the LOI ceases and discourages self-questioning and self-reflection when ESL learners are expected to narrate their writing in a language they neither speak fluently nor understand. Their communication skill does not develop and they do not engage in critical reflection and inquiry regarding their experiences, thus they are unable to think creatively, integrate meaningfully prior and new knowledge, explore and ponder on their real-life experiences. They also fail to develop a deeper understanding of their experiences and create unique innovative solutions to complex real-life problems (Kirui et al. 2017). Thus, ESL learners are challenged to construct their own meaningful learning and develop self-awareness through critical reflections. The dire consequence in nursing is that they fail to appropriately express and share their emotions and feelings as well as convey knowledge and complex messages relating to their clients to other members of the multidisciplinary team (Scherer 2016).

### Theme 4: Language incompetence undermined learners’ self-esteem

The participants stated that learners had difficulty engaging with fellow learners during dialectic dialogic activities such as argumentation because of lack of self-esteem owing to lack of command of the LOI. Moreover, engaging in group discussions and argumentation became embarrassing for these learners. According to Yilmaz, Sabancıoğulları and Kumsar (2016), high self-esteem affords learners a platform to acquire mastery of a task and develop higher order thinking skills through effective communication. For learners to succeed academically, they need to believe that they are competent to master a task within a psychologically safe environment because mastery develops self-confidence, academic efficiency and high self-esteem (Ndawo 2017). In a psychologically unsafe environment brought by a lack of command of the LOI, ESL learners experience stress, fear and anxiety as well as lose their self-esteem and self-confidence. They also feel incompetent to participate in exploration of extensive literature, research and use of evidence-based practices and thus disinclined to improve and meet their academic goals. They lack trust in their reasoning, therefore do not make mistakes and take intellectual academic and emotional risks for fear of being ridiculed resulting in stifled academic growth (Scherer 2016).

The participants also experienced silence and passivity from ESL learners in their learning environments during group discussions. English-as-a-Second Language learners experience uncertainty and doubt about the accuracy of their statements and facts as well as a lack of ownership and pride in their work because of the limited LOI proficiency. When their self-esteem and self-confidence are negatively affected, ESL learners do not become active contractors of their own knowledge and that retards their ability to learn beyond their potential (Sharif 2013). Similarly, Schruijer (2016) reported that ESL learners will intentionally choose not to share their knowledge aloud and respond to challenging questions during group discussions. Their passivity is deliberate and serves to allay their own anxieties and stress relating to the use of the LOI, thereby protecting their self-esteem against any threats. Their meaningful engagement and social interactions are limited, which results in lack of critical discourse and sharing of intellectual capital that may result in a unilateral perspective of a learning activity. This limitation further retards meaningful learning, open-mindedness and development of effective communication skill required of a nursing graduate in the 21st-century healthcare setting (Kivunja 2015; Ndawo 2017). In addition, ESL learners are unable to achieve their learning objectives and envisaged academic goals.

The participants also noted that their ESL learners did not engage in constructive pedagogical strategies such as argumentation because of low self-esteem. This results in those few learners who are fluent in the LOI dominating the argumentation session, resulting in an undemocratic participation, while the non-fluent learners have difficulty listening critically, following the logic of the arguments, formulating strong counterarguments and expressing themselves eloquently in a way that they make sense and understood. Thus, co-creation of meaningful diverse learning is hindered. Yet, argumentation in the language that learners understand makes them believe that they are competent and can master the art of argumentation because they can express themselves well (Scherer 2016). That way argumentation as a pedagogical strategy becomes academically developmental.

## Limitations

The contextual nature of the study limits the generalisation of the findings to other nursing colleges; however, the study provides rich information of the nurse educators’ experiences on the LOI as a barrier to the facilitation of academic activities. Even though the study was about teaching, learning and assessment, learners’ experiences of LOI were not explored in this study because the study was limited to nurse educators’ experiences. Only ESL nurse educators were invited to participate in the study and in hindsight other nurse educators should have been invited to participate with regard to the influence of LOI on the facilitation of learning only in ESL learners.

## Recommendations

### Nursing education

While policies in higher education institutions have not changed regarding the use of English as a LOI, it is recommended that nurse educators engage in self-reflection about the effectiveness of their pedagogical approaches that are influenced by this language. They must actively reflect on their beliefs that might misconstrue learners’ behaviour and language use, thereby limiting effective teaching and meaningful learning (Lucas, Villegas & Martin 2015). Furthermore, they should create a learning environment that is emotionally supportive, non-threatening, non-judgemental and safe to make language mistakes, thereby ensuring that critical thinking is encouraged. To assist with the development of proficiency in the LOI, the participants stated that ESL learners must be encouraged to speak in their indigenous language and those learners who are proficient in LOI to assist with translation to retain meaning. Dialogues, critical discourse and argumentation in small heterogeneous groups with more linguistically able learners should be encouraged while simultaneously providing homework that stimulates self-directed learning for ESL learners. Learners should be encouraged to use blogs, Twitter, electronic Journals and Portfolios daily to facilitate comprehension and acquisition of the LOI while improving their professional learning. Old assessment papers should be made available to learners so that they can learn to interpret questions and understand what is required of them without answering such papers. However, this exercise must be given sufficient time for learners to immerse in these papers meaningfully.

### Nursing practice and research

The researcher recommends that following the experiences of nurse educators, learners’ experiences on how English as the LOI influences their academic performance should also be explored and recommendations be made based on the findings. The impact of lack of proficiency in the LOI on learners’ academic performance in this nursing college should be investigated. Furthermore, the impact of lack of proficiency in the LOI on learners’ clinical practice should also be investigated.

## Conclusions

This article explored and described the experiences of nurse educators regarding the influence of LOI in the facilitation of academic activities. The findings suggested that academic activities, namely teaching, learning and assessment, are negatively influenced when English is used as a LOI for ESL educators and learners. These findings add to the body of existing literature on the influence of LOI as a second language in academic activities. Even though LOI is used by ESL nurse educators and learners to engage in dialogic learning activities and to collect, organise, analyse, critically think about and evaluate information while communicating with significant others and within relevant communities, when there is limited proficiency there is lack of creative and meaningful teaching, learning and assessment that takes place. The article concluded with recommendations on nursing education, nursing practice and nursing research on improving LOI proficiency in ESL nurse educators and learners.
